# DNA damage in preserved specimens and tissue samples: a molecular assessment

**DOI:** 10.1186/1742-9994-5-18

**Published:** 2008-10-23

**Authors:** Juergen Zimmermann, Mehrdad Hajibabaei, David C Blackburn, James Hanken, Elizabeth Cantin, Janos Posfai, Thomas C Evans

**Affiliations:** 1 New England Biolabs Inc., 240 County Rd., Ipswich, MA, 01938, USA; 2 Canadian Centre for DNA Barcoding, Biodiversity Institute of Ontario, University of Guelph, Guelph, Canada; 3 Department of Organismic and Evolutionary Biology and Museum of Comparative Zoology, Harvard University, 26 Oxford Street, Cambridge, MA 02138, USA

## Abstract

The extraction of genetic information from preserved tissue samples or museum specimens is a fundamental component of many fields of research, including the Barcode of Life initiative, forensic investigations, biological studies using scat sample analysis, and cancer research utilizing formaldehyde-fixed, paraffin-embedded tissue. Efforts to obtain genetic information from these sources are often hampered by an inability to amplify the desired DNA as a consequence of DNA damage.

Previous studies have described techniques for improved DNA extraction from such samples or focused on the effect of damaging agents – such as light, oxygen or formaldehyde – on free nucleotides.

We present ongoing work to characterize lesions in DNA samples extracted from preserved specimens. The extracted DNA is digested to single nucleosides with a combination of DNase I, Snake Venom Phosphodiesterase, and Antarctic Phosphatase and then analyzed by HPLC-ESI-TOF-MS.

We present data for moth specimens that were preserved dried and pinned with no additional preservative and for frog tissue samples that were preserved in either ethanol, or formaldehyde, or fixed in formaldehyde and then preserved in ethanol. These preservation methods represent the most common methods of preserving animal specimens in museum collections. We observe changes in the nucleoside content of these samples over time, especially a loss of deoxyguanosine. We characterize the fragmentation state of the DNA and aim to identify abundant nucleoside lesions. Finally, simple models are introduced to describe the DNA fragmentation based on nicks and double-strand breaks.

## Introduction

Preserved tissue samples and museum specimens are a vast repository of genetic information of interest to biological and medical researchers. These samples are important to cancer biopsy tissue research, forensic investigations and phylogenetic studies based on museum specimens, including extinct species. A recent review outlines important considerations and guidelines when working with specimens from museums and other natural history collections [1].

DNA is repaired with great efficiency in living cells [2], but this repair ceases upon death of the organism or preservation of a sample. Depending on the conditions of storage, the DNA in such samples degrades more or less strongly over time and often becomes inaccessible to genetic studies [3-6] (but see also [7,8]).

Formaldehyde is a commonly used preservative for field collected specimens and cancer biopsy tissue [9,10]. Tissue biopsies are typically stored as so-called formaldehyde-fixed paraffin-embedded (FFPE) samples. FFPE's are prepared by "dipping" the sample in a 3.7% formaldehyde solution for up to 24 h. In recent years, it has become common practice to use a formaldehyde solution buffered to pH 7.0 [11]. The unbuffered solution has a pH of ~4.5. Such a drop in pH would lead to an increased rate of DNA depurination. Samples will then be embedded in paraffin for storage.

The reaction of formaldehyde with nucleic acids has been studied in great detail. One of the earliest reports was published by Feldman in 1973 [12]. A number of reaction products were reported but the main adduct observed is the addition of a hydroxymethyl-substituent to primary and secondary amine groups of the respective base. These investigations were continued in a series of papers by von Hippel and coworkers who describe the reactions of formaldehyde with free bases and a number of aromatic amines, both for exocyclic amino and for endocyclic imino groups [13-16]. Again, the hydroxymethyl-adduct was reported to be the main reaction product. The reaction mechanism was investigated ab initio by Chang et al. and found to be most likely base-catalyzed [17]. The consequences of tissue preservation with formaldehyde on the integrity of the extracted DNA have been described in a number of studies, see for example Lit. [18-21]

Many museum specimens, particularly insects, are stored pinned and are not subjected to any further preservation treatment [22]. While the exoskeleton of the insects is stable over many years, the soft tissue soon dries out and decomposes. In a recent study, the effect of different methods of killing and specimen storage on mitochondrial DNA content and PCR success from *Drosophila simulans *specimens was described [23]. The study showed a significant impact of storage time on PCR success, whereas the method of killing and the investigated storage conditions had no marked effect. Main factors affecting DNA during storage are expected to be partial dehydration and exposure to air and light, all potentially leading to diverse types of damage. The deamination of cytidine residues has been identified as a common miscoding lesion in studies of ancient DNA [24].

In this study, our goal was to characterize on the molecular level the damage present in DNA samples from tissues of preserved animal specimens. We use PCR-based assays to some extent as a measure of usability of samples, but mainly focus on the molecular characterization of the DNA composition and the characterization of individual lesions from genuine DNA samples.

Furthermore, we have developed two models to describe DNA fragmentation by nicks and double-strand breaks and compare our data to these models.

## Materials and methods

### Specimens

All moth specimens belong to the species *Euxoa messoria*. They were collected over a 45-year period (Table 1) and were preserved pinned with no additional preservative. Specimens of three different frog species (Table 2) were collected as part of ongoing research unrelated to this study and preserved using standard methods (e.g., Lit. [25]). Frogs were killed using an aqueous solution of chloretone and, for adult frogs, a sample of liver tissue was preserved in 95% ethanol. Adult specimens were then fixed in 3.7% neutral-buffered formaldehyde overnight and then transferred to 70% ethanol for long-term storage. In large specimens (e.g., *Astylosternus*), a small volume (~1 milliliter) was injected into the body cavity during fixation. For tadpoles, a small piece of tail (mostly muscle) was excised and stored in 95% ethanol; following common practice, the remaining specimen was fixed and stored in 3.7% formaldehyde. Animal care procedures are approved by the Harvard University/Faculty of Arts and Sciences Standing Committee on the use of Animals in Research and Teaching. An Animal Welfare Assurance statement is on file with the university's Office for Laboratory Welfare (OLAW).

**Table 1:** Origin and preservation of frog tissue samples

No.	Frog: Order and Species	MCZ No.	Stage	Preservation	Date	Location
1a	*Leptodactylodon axillaris*	136883	adult	E	25-Sep-04	Mt. Bamboutos, Cameroon
1b		136883		F, E		

2a	*Astylosternus rheophilus*	136934	adult	E	25-Sep-04	Mt. Manengouba, Cameroon
2b		136934		F, E		

3a	*Afrana angolensis*	136997	adult	E	16-Jan-05	Lukhubula River, Malawi
3b		136997		F, E		

4a	*Afrana angolensis*	136998	adult	E	16-Jan-05	Lukhubula River, Malawi
4b		136998		F, E		

5a	*Astylosternus rheophilus*	137937	adult	E	13-Jul-06	Mt. Manengouba, Cameroon
5b		137937		F, E		

6a	*Astylosternus rheophilus*	not acc.	tadpole	E	13-Jul-06	Mt. Manengouba, Cameroon
6b		not acc.		F		

7a	*Leptodactylodon axillaris*	137972	adult	E	08-Aug-06	Mt. Bamboutos, Cameroon
7b		137972		F, E		

8a	*Leptodactylodon axillaris*	not acc.	tadpole	E	08-Aug-06	Mt. Bamboutos, Cameroon
8b		not acc.		F		

(not acc.: not accessioned)

**Table 2:** Collection date of moth specimens

Moth specimens	
Sample No.	Collection date

1–4	August 2005
5–9	August 2000
10–13	September 1995
14–17	September 1990
18–19	September 1984
20–23	August 1980
24–27	September 1977
28–31	August 1974
32–37	September 1970
38–42	September 1965
43–47	August 1960

After returning from the field, tissue samples in 95% ethanol were stored at -80°C. For this study, another piece of the same tissue (i.e., liver or tail) was excised from the whole preserved specimens; these tissue samples were transferred to 95% ethanol. To qualitatively evaluate the effect of storage time and reduce the effect of species or developmental stage on our results, we analyzed tissues from adults collected over two different years, as well as tadpoles of the same species. Those samples that were stored or fixed using formaldehyde will be referred to as *exposed *to formaldehyde.

### DNA extraction

Small aliquots of frog tissue (1–3 mg) were obtained from the preserved specimens in March 2007. The tissue was lysed and DNA was purified using the DNeasy kit (Qiagen) following the manufacturer's protocol. Extracted DNA was stored in TE buffer at 4°C.

A leg from each moth specimen was used for DNA extraction, using the NucleoSpin96 kit (Macherey-Nagel). Elution was performed with 40 μl water. The eluate was stored at -20°C.

### Fragment analysis by capillary electrophoresis

An aliquot of 1–5 μl of extracted DNA was labeled with Fluorescein-12-ddATP (PerkinElmer, Boston, MA) using Terminal Transferase (NEB, Ipswich, MA) according to the accompanying protocol, resulting in a 10 μL reaction volume. The reaction was incubated at 37°C for 1 h, then applied to a Centri-Sep column (Princeton Separations) [26].

For the removal of terminal phosphates on the DNA fragments, aliquots of 3 μl DNA were treated with 5U Antarctic Phosphatase (NEB) in a total reaction volume of 10 μl. The reaction was incubated at 37°C for 1 h, followed by inactivation of the phosphatase at 65°C for 5 min. This was followed by labeling with TdT as described above.

**Figure 1:**
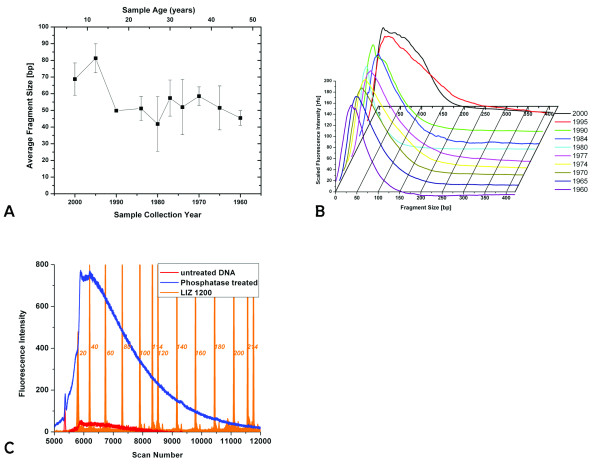
**Panel A shows the size distribution of DNA extracted from moth samples.** See methods for details of size determination. Panel B shows the raw data of the FAM-labeled DNA fragments, averaged for each year. Data were scaled to the same height for comparison. Note the decrease in peak width with sample age. Panel C shows the raw data obtained for moth sample 3 from a Capillary Electrophoresis run. Labeling the DNA without any prior treatment results in the fragment distribution shown here in red. An aliquot of the same sample was treated with Antarctic Phosphatase before the TdT labeling reaction, shown in blue. The size distribution of the fragments does not change, while the intensity is increased by a factor of 2–15 for different samples. The LIZ1200 size standard is shown in orange, numbers indicate the fragment size in bases.

An aliquot of 1–2 μl of the eluate was mixed with 9 μl Hi-Di (Applied Biosystems) and 0.5 ml GENESCAN LIZ1200 size standard (Applied Biosystems). Samples were analyzed on a 3130xl Genetic Analyzer (Applied Biosystems), using a 36 cm array, POP7 polymer, an injection time of 10 s and a total run time of 6200 s. An example of the raw data used for fragment size determination is shown in Figure 1c for moth sample 3.

Raw data were imported into Origin7.5 (Microcal) for detailed analysis. For the determination of the most abundant fragment size of a sample, the data curve for the FAM fluorescence was subjected to smoothing, using the adjacent average method over 500 points. The smoothed curve was fitted to a peak function, equation 1, to determine the position of the maximum (in scan numbers).

(1) $$\begin{array}{l} y=y_{0}+Ae^{\left(-e^{\left(-z\right)}-z+1\right)}\\ z=\left(x-xc\right)/w \end{array}$$

*w: width, xc: center, y*_0_*: offset, A: Amplitude*

To convert this into base pairs, the elution times of the size standard fragments (in scan numbers) were plotted against the known size of each fragment of the LIZ1200 standard and fitted to a sigmoidal growth curve, equation 2.

(2) y=A−1A21+e(x−x0)/dx+A2

*A*_1_*: initial value, A*_2_*: final value, x*_0_*: center, dx: time constant*

The fitting result for the size standard together with the peak of the FAM fluorescence were used to determine the most abundant size of DNA fragments in a given sample. For the distribution of fragment sizes, the peak width (full width at half height) was used, as determined from the fit of equation 1.

For quantitation of total DNA content, a baseline was fitted to the total FAM signal, the signal was then integrated using this baseline. As a test for the linearity of detection in our CE, we used the Φ X174 DNA ladder (NEB) in a serial dilution. We found a linear correlation between data integral and sample concentration in the range of 2–20 ng/μl (R = 0.997, data not shown).

### DNA digestion

Extracted DNA was digested using a published method [27] with modifications. Aliquots of 1–10 μl of extracted DNA were incubated with 1 μl DNase I (2U/μl, NEB), 10 μl Snake Venom Phosphodiesterase (0.26 mU/μl, Sigma-Aldrich) and 2 μl Antarctic Phosphatase (5U/μl, NEB) at 37°C overnight. Using this procedure, unmodified DNA was completely digested to the mononucleoside level as judged by HPLC (data not shown).

### HPLC separation

Digested DNA samples were analyzed on an Agilent 1100 HPLC system equipped with a Develosil RP-Aqueous C30 column (Nomura Chemical Co.). Solvent A was MilliQ water containing 1% (v/v) formic acid and solvent B was gradient grade methanol containing 0.25% (v/v) formic acid. An elution profile was used of 2–20% B over 30 min increasing to 98% over another 20 min then 98% B for 10 min and finally returning to 2% B over 20 min. The flow rate was set to 20 μl/min and the eluate monitored at 254 nm. Typically, 4 μl of each sample were injected using the well-plate sampler.

### Mass spectrometric analysis

For mass spectrometric analysis the HPLC system described above was connected directly to the sample inlet of an Agilent ESI-TOF mass spectrometer. Mass spectral data were recorded in positive ion mode over the entire duration of the HPLC run. Data were analyzed using Analyst QS (Agilent).

### Pulsed field agarose gel electrophoresis

For the detection of large DNA fragments, aliquots of the frog DNA were loaded on a 1% agarose gel and separated over 15 h with a switch time from 1–12 s and a voltage of 6 V/cm. The marker was PFG marker N0350 (NEB).

### PCR

A 500 bp piece of the *Euxoa messoa *barcode sequence was amplified using primers pJZ-moth1-se TTAGGTAATCCAGGATCTTTAATTG and pJZ-moth1-as ATGATAATAATAATAAAAATGCAGT. Amplification was performed with Taq DNA polymerase (NEB), with an initial denaturation at 95°C for 2 min., then 30 cycles of 95°C for 15 s, 55°C for 10 s, 72°C for 30 s, and a final extension at 72°C for 5 min.

Primer sequences for the PCR of frog mitochondrial 16S ribosomal RNA correspond to those of Darst and Cannatella [28]. The primers for the first exon of the nuclear gene for rhodopsin are ACGGAACAGAAGGTCCCAAC (5' primer) and AGCGAAGAAGCCTTCAAAGT (3' primer). PCR reactions were carried out with Phusion DNA polymerase (NEB), with initial denaturation at 98°C for 30 s, then 30 cycles of 98°C for 10 s, 60°C for 10 s, 72°C for 45 s, and a final extension at 72°C for 10 min.

### Modeling of DNA nicking

An algorithm was written in C to simulate fragmentation of double-stranded DNA by repeated nicking events. The simulation required four input parameters: simulated time period length (*t*) in years, DNA size (*L*) in megabases, nick rate (*n*) in nicks per megabase per day, and proximity of opposite strand nicks (*p*) that result in a double-stranded break given in bases. The program initiates the C library random number generator function so that repeated calls to the generator will return uniformly distributed random integers between 1 and 2*L*10^6^. Random number r will represent a nick on the rth position of the forward strand if *r *<L*l0^6^, otherwise the program assigns the nick at position *rc *= *r *- L*l0^6 ^on the reverse strand. The imaginary sequence is "nicked" n*L*365*y times at positions indicated by the random numbers returned from consecutive calls to the random number generator. Next the program identifies where opposite-strand nicks occur within *p *bases, and records double-stranded breaks. Distances between consecutive breaks, measured on the forward strand of DNA, give fragment lengths. These are tabulated and reported in a size-sorted list. The simulation is run with different combinations of input parameters.

## Results and discussion

### I. Moth specimens

The analyzed moth specimens all belonged to the species *Euxoa messoria*, a native of North America. The specimens were preserved pinned and not exposed to other preservatives prior to DNA extraction.

The fragmentation state of the extracted DNA was evaluated by Capillary Electrophoresis. There is a general correlation between the age of the sample and the fragment size, which gets smaller with increasing age of the sample (Figure 1).

The samples from 2000 show a most abundant fragment size of ~70 bp. The raw data show a considerable spread of sizes, ranging from approximately 20 to 170 bp. All samples from 1990 and older have a most abundant fragment size of approximately 50 bp. The distribution of fragment sizes becomes narrower with sample age (see Figure 1b) and is in the range of 20–100 bp for the oldest samples from 1960. Within the storage period investigated here, this appears to be a semi-stable fragment size. Notably, there is a small increase in fragment size for the samples between 30 and 40 years of age (collected around 1970). The reason for this slight deviation from the general trend is not clear.

We were not able to determine the size distribution for the youngest samples from 2005. We assume that DNA in these samples is too big to enter the capillary or elute within the observed time. On the other hand, the DNA concentration was too low to be visualized by Pulsed Field Agarose gel electrophoresis. The successful PCR amplification from these young samples (see below) corroborates our assumption of the presence of large fragments. Fragments too small to be detected by capillary electrophoresis or a general lack of DNA would not lead to a PCR product.

Analysis by fluorescent labeling followed by Capillary Electrophoresis will only show the more abundant fragments. While the most abundant fragment size lies within the range described above, there may be larger fragments present in amounts sufficient for PCR amplification, but too low for detection with this methodology. This seems to be the case for the samples from 2000, for which a fragment distribution of 20–170 bp is observed, yet a 500-bp product can be amplified from these samples. Alternatively, the 500-bp amplicon may be assembled during PCR from smaller template fragments. This seems more unlikely in light of the unsuccessful amplification from older samples, which contain fragments of comparable size to the year 2000 samples.

The CE setup used in this study is limited to fragment sizes from 20 to ~1500 bp. Larger fragments would escape detection. While the presence of large fragments cannot be excluded based on our experiments, the accumulation of two fragment populations that are very distinct in size appears unlikely and to our knowledge has not been reported before. Hence we conclude that the fragment sizes observed via CE give an appropriate representation of the DNA recovered from the moth tissue.

The fragment analysis is based on DNA labeling with FAM-ddATP and Terminal Transferase. This reaction requires the presence of a free 3'-hydroxyl group on the fragments. If fragmentation occurred after formation of an abasic site, two main mechanisms were described for the ensuing chain break, either a β-elimination or the formation of a 3'-4'-cyclic phosphate [29,30], neither resulting in a free 3'-hydroxyl. However, more complex mechanisms have been suggested [31], and the successful labeling is proof of the existence of such groups. It is possible that only a small portion of the fragments present in the DNA extract have a free 3'-hydroxyl group, but we assume the underlying mechanisms of fragmentation to be sequence independent, but see for example Ref. [32], and hence yield a statistical representation of fragment sizes resulting from different mechanisms. We found that treatment of the DNA samples with Antarctic Phosphatase prior to labeling improved the amount of labeled DNA by a factor of 2–10. (Figure 1C)

**Figure 2:**
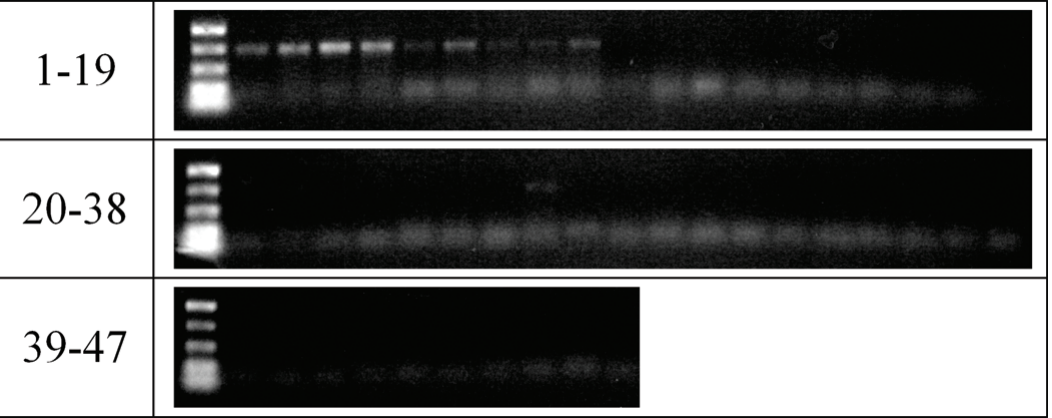
**PCR of a 500-bp part of the moth barcode sequence**. An amplicon was only obtained for the samples collected in 2005 and weakly for samples from 2000. All other reactions show only primer dimers.

PCR amplification of a 500-bp segment of the cytochrome oxidase I (coxI) gene was successful for the youngest samples, dating from 2005 and 2000 (Figure 2). However, no amplicon was obtained for the older samples. We found a seven-fold decrease in the amount of extractable DNA as the samples increase in age (data not shown). Furthermore, as discussed above there is a decrease in average extracted DNA size. A third possible issue is the presence of base lesions that interfere with PCR.

**Figure 3:**
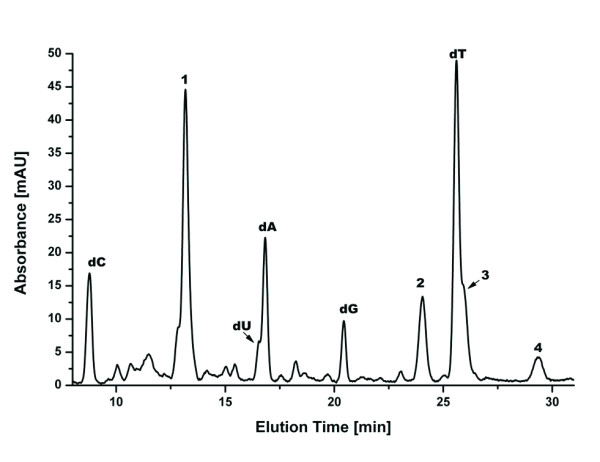
**UV-Chromatogram of Moth specimen 30, collected in 1974**. Digest of a moth sample, showing the four standard deoxynucleosides and several additional components. The numbers 1, 2, 3, 4 (exact masses 245.07(5), 380.03(4), 367.17(6) and 355.18(7) Da, respectively) label components of the DNA preparation not yet identified. These are potential lesions preventing PCR amplification from these samples. They are not present in the youngest specimens.

We attempted to address whether base lesions could be a problem in DNA amplification from these museum samples by using the LC-MS analytical technique. The moth DNA was digested to nucleosides and applied to an LC-MS. Figure 3 shows the UV chromatogram for the digest of a sample collected in 1974, sample 30. In the chromatogram the four deoxynucleosides can be readily identified based on their elution times and masses. There is also some indication of the presence of deoxyuracil. This may be due to the presence of a cytosine deaminase in the DNase I preparation and hence would be an artifact of sample preparation [Zimmermann J: **unpublished results**]. Four other significant peaks have been labeled 1, 2, 3 and 4 with exact masses of 245.07(5), 380.03(4), 367.17(6) and 355.18(7) Da, respectively. Based on their mass alone it has so far not been possible to identify these compounds with confidence. Work to identify them using MS/MS techniques is in progress.

Interestingly, when comparing the UV chromatograms for all the moth samples, it became apparent that the peak for dG became smaller for the older samples, while peaks for the other nucleosides remained largely similar in intensity. The dG peak can only be detected in the UV chromatograms of the youngest samples. The dG ion can be extracted from the Total Ion Current of the mass spectrometry run for each sample as an Extracted Ion Chromatogram (XIC), to give a more accurate picture. In this way, dG can be detected in all but the samples older than 1970. No peak for dG is detectable in the mass spectra of the oldest samples from 1965 and 1960.

The absolute area of the individual peaks depends on the sample concentration, which differs between the different extracts of moth DNA. A determination of the concentration by measuring the A_260 _values was not attempted due to the small available sample amounts. This makes it impossible to compare dG contents of different samples by a direct comparison of peak areas. We therefore consider the ratios of peak areas within each sample, namely the area ratios of dA/dT and of dG/dC. While the numerical value of these ratios has no physical meaning, it is expected to remain constant if the base composition of the different samples remains the same. This is expected for DNA samples from the same species, assuming no DNA degradation.

**Figure 4:**
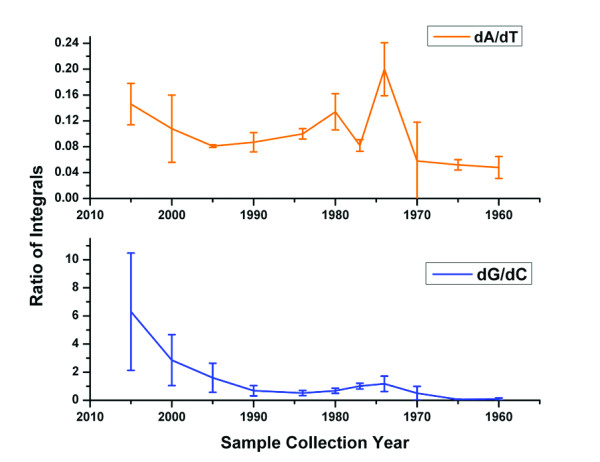
**Estimate of dG content in moth DNA**. All moth samples were subjected to LC-MS analysis. For the software analysis, ions of the four deoxynucleosides were extracted from the total ion currents and resulting peaks were integrated. The plot shows the ratios of integrals for dA/dT and dG/dC, respectively. While the dA/dT ratio stays fairly constant over time, the dG/dC ratio drops very much. Indeed, dG cannot be detected in the oldest moth samples.

We do observe a reasonably constant value for the dA/dT ratio (Figure 4a). There is some variation between years, and a relatively large bandwidth of values within samples from a given year, nonetheless the ratio stays mainly around a value of ~0.10. In contrast, the dG/dC ratio declines rapidly from a value of ~6 to ~1 during the first 15 years of sample preservation and remains low for the older samples (Figure 4b). As seen above for DNA fragmentation (Figure 1a), there is a small increase in the ratio for the samples from around 1970.

Taken together, the observation of the diminishing peak for dG in the mass spectrometric analysis, the persistence of peaks for the three other nucleosides, and the decreasing ratio of the dG/dC peak areas show a striking effect of storage time on the dG content of DNA in these samples. The fate of the dG residues is currently under investigation. At this point we do not know whether the loss of dG is due to depurination and the creation of an abasic site, or to a specific base modification. A combination of the two processes seems possible, in which certain chemical modifications of the base lead to an increased rate of depurination of the modified residue versus normal dG. The resulting abasic site is prone to hydrolysis and would thus facilitate fragmentation of the DNA. A commonly observed oxidative lesion is 8-oxo-deoxyguanosine. We analyzed selected samples for this compound by creating an XIC from the ESI-TOF data, searching for ions with a mass of 284 – 285 m/z (exact mass of 8-oxo-dG: 283.09 Da). We did not find the compound with this strategy and conclude that, if present, the amounts of 8-oxo-deoxyguanosine in the extracted and digested DNA samples must be too low to be detected in this way. Using the same approach, we also searched for 7-Hydro-8-oxo-deoxyguanosine (Fapy-desoxyguanosine, exact mass: 285.11 Da), a compound sometimes found in samples containing 8-oxo-dG and a possible reaction product of 8-oxo-deoxyguanosine. Again, we did not find this compound.

### II. Frog specimens

**Figure 5:**
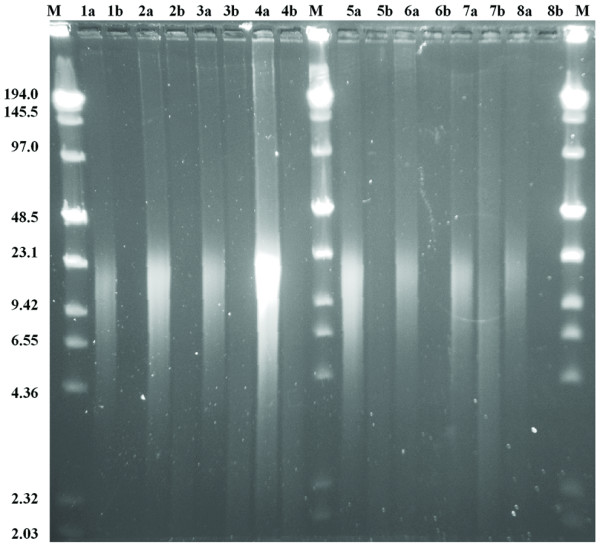
**Pulsed Field Agarose Gel of extracted frog DNA**. Only the samples preserved in ethanol alone show detectable levels of DNA in this gel. The average size of the fragments is ~18 kbp. For the formaldehyde preserved samples, only sample 7b shows a detectable smear of DNA.

At the turn of the last century, formaldehyde came into wide usage for preservation of biological material. Exposing or simply maintaining a specimen in formaldehyde had the benefit of preserving sample morphology much more effectively than ethanol alone. Unfortunately, formaldehyde inhibits modern genetic analytical techniques such as PCR and DNA sequencing. This may be due to formaldehyde-induced crosslinks or adducts or to the fact that formaldehyde solutions need to be periodically buffered with phosphate buffer to prevent a precipitous drop in pH due to formic acid formation. Although these formaldehyde effects are all problematic, work is on-going to determine the specific problem. We therefore chose to analyze actual museum specimens that had been exposed to formaldehyde during preservation. Because of an unrelated research project, it was possible to obtain tissue samples from individual specimens that had been preserved using different combinations of formaldehyde treatment and ethanol (Table 2). For two species, samples were available that were collected during different field seasons thus enabling us to evaluate at the short term effect of storage in ethanol or formaldehyde. For tissues from a given specimen, all samples were of the same tissue (liver or tail musculature), approximately equal size, and prepared by the same person (DCB). The result of the tissue lysis and concentrations of extracted DNA (as determined from the A_260_) are given in table 3. As was later found by LC analysis (see below), the samples contain significant amounts of RNA. The concentrations given in the table hence are the sum of DNA and RNA in each extract. Samples were first characterized by Pulsed Field Gel electrophoresis (Figure 5). The average fragment size from specimens preserved only in ethanol is approximately 18 Kbp, with a range from approximately 11 to 23 Kbp. DNA extracts from formaldehyde-exposed specimens show no detectable DNA on this gel, with the exception of sample 7b, which shows a weak smear.

**Table 3:** Concentration of extracted frog DNA

No.	Preservation	DNA conc. [ng/μl]
1a	E	93.4
2a	E	58.1
3a	E	19.1
4a	E	93.7
5a	E	36.9
6a	E	25.1
7a	E	127.3
8a	E	36.4
6b	F	2.8
8b	F	1.6
1b	F, E	11.6
2b	F, E	21.1
3b	F, E	18.6
4b	F, E	18.6
5b	F, E	10.7
7b	F, E	61.9

**Figure 6:**
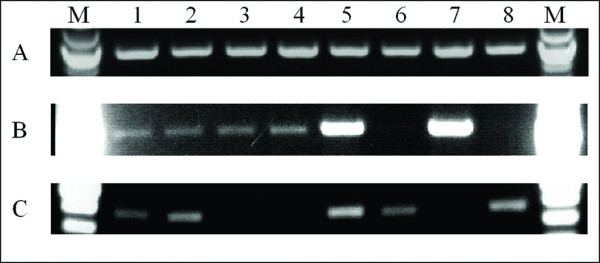
**PCR using extracted frog DNA**. Panel A shows amplification of a 1,000-bp segment of the mitochondrial 16S gene from ethanol preserved samples. (sample 1a-8a). Panel B shows the same amplification from formaldehyde-exposed samples (sample 1b-8b). Panel C shows the amplification of a 500-bp segment of the nuclear rhodopsin gene (sample 1a-8a) from ethanol preserved samples. No product was observed for the rhodopsin gene with samples 1b-8b, which were exposed to formaldehyde during preservation.

The samples were used as templates for the PCR amplification of one mitochondrial and one nuclear gene fragment. Amplification of the mitochondrial 16S ribosomal RNA gene was successful for all samples preserved only in ethanol (Figure 6, panel A). The same experiment showed mixed results for the formaldehyde-exposed samples. Samples 1b-4b yielded a very low amount of product, barely visible on the gel. Samples 5b and 7b yielded a much higher amount of product and were collected more recently. Samples 6b and 8b show no detectable product on the gel; both specimens are tadpoles that were stored in 3.7% formaldehyde for approximately a year before tissues were excised and transferred to 95% ethanol for these analyses.

The formaldehyde-exposed samples giving small to good product amounts were only fixed in formaldehyde and then transferred to ethanol. Of these, the youngest two samples 5b and 7b (i.e., those preserved for the shortest time) yield the largest amount of product. Samples 6b and 8b were preserved only in formaldehyde since their collection and no product can be obtained from them. As was described previously [33], there is a clear negative correlation between exposure time to formaldehyde and success of PCR.

Amplifications of the nuclear rhodopsin gene were more difficult to achieve from these samples. This is clearly due to the abundance of the mitochondrial gene at several hundred or thousand copies per cell, as opposed to only two copies for each nuclear gene.

The rhodopsin sequence can be amplified for samples 1a, 2a, 5a, 6a and 8a, but not for the remaining samples 3a, 4a and 7a. As there is no obvious correlation between the ability to amplify this nuclear gene and the preservation, sample age, or developmental stage, the variation in these results is most likely stochastic in nature. They may reflect small differences in the actual process of sample preservation, tissue morphology (i.e., ease or difficulty of tissue lysis and DNA extraction from different tissues) and variations in DNA yields during the extraction process.

In our hands it was not possible to amplify the rhodopsin target sequence from the formaldehyde-exposed tissues.

**Figure 7:**
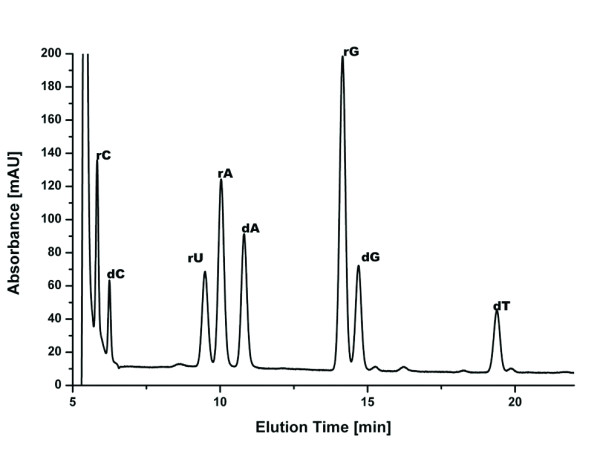
**UV-Chromatogram of digested frog DNA, sample 1a**. RNA had not been completely removed before digestion, "r" denotes ribo-nucleosides, "d" denotes deoxy-nucleosides.

**Figure 8:**
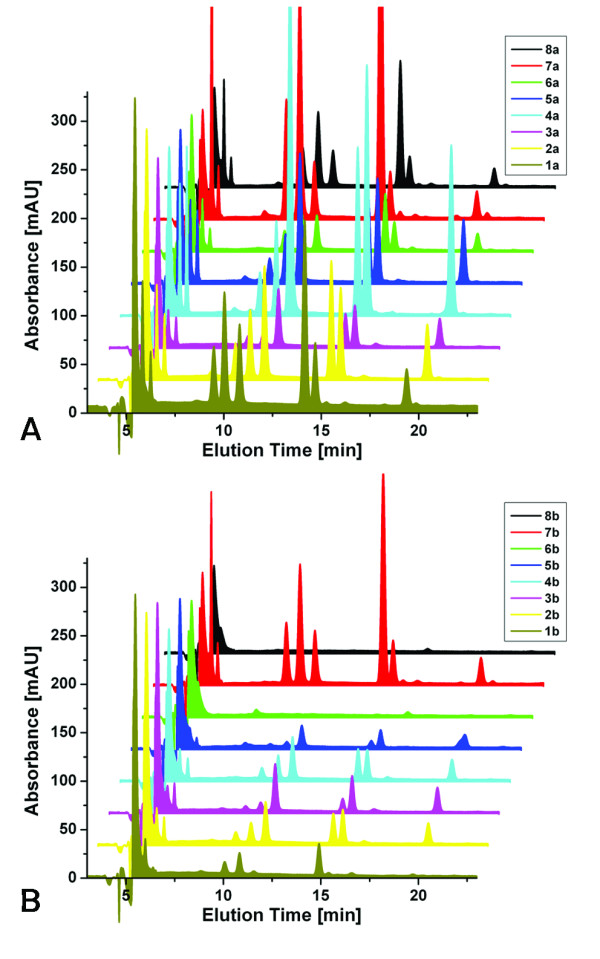
**UV-Chromatograms of all frog samples**. Panel A shows those samples preserved only in ethanol. The overall concentration of extracted DNA varies somewhat, but no extra peaks are detected in these samples. Panel B shows those samples preserved using formaldehyde. The overall yield of DNA is considerably lower, using tissue samples of comparable weight. Additional peaks, which would indicate potential lesions or formaldehyde adducts, are not apparent.

Aliquots of the frog DNA were digested to the nucleoside level, separated by HPLC, and components identified by ESI-TOF-MS. A representative UV chromatogram is shown in Figure 7 for sample 1a. The four standard deoxynucleosides can readily be identified based on their retention time and mass. This chromatogram also shows significant amounts of ribonucleosides, showing that RNA was not digested completely before DNA purification and inadvertently copurified in the DNA extraction process. Figure 8 shows comparisons of UV chromatograms for the ethanol-preserved samples as well as for the formaldehyde-exposed samples. There is some variation in the overall amount of extracted DNA in each of the two groups. In comparison, the amount of DNA extracted from the formaldehyde-exposed specimens is strikingly lower than that from ethanol-preserved specimens. New peaks, hinting at the formation of adducts in either group, are not apparent. It is currently unclear whether the DNA is crosslinked within the tissue and cannot be extracted, or is degraded heavily by the formaldehyde treatment, in particular the concomitant drop in pH, which leads to an increased rate of depurination/depyrimidination.

As expected, differences between the different frog species or between different developmental stages were not observed in these experiments.

The main obstacle in obtaining DNA from formaldehyde-preserved samples appears to be the early stage of DNA extraction, rather than specific lesions that inhibit PCR amplification. We performed tests with mouse liver tissue, which was preserved in formaldehyde for different lengths of time, mechanically homogenized and then lysed by treatment with a standard lysis buffer and Proteinase K at 55°C. This treatment leads to complete solubilization of fresh tissue within one hour for a piece of tissue of ~100 mg. In contrast, tissue fixed with formaldehyde for as short as a few minutes will not dissolve completely even after several days of incubation at 55°C, repeated spiking with Proteinase K or use of increased concentrations of chaotropic agents, such as 8 M guanidinium. We suggest that most of the DNA in samples preserved in formaldehyde is crosslinked intricately to the surrounding tissue components and cannot be extracted by standard DNA extraction methods.

Numerous lesions in DNA exposed to formaldehyde have been described in studies using nucleotides or isolated DNA [34,35]. Presumably, similar lesions occur in whole tissue. This was recently demonstrated for the first time for DNA from rats [36]. The animals were treated with N-nitrosodimethylamine or 4-(methylnitrosamino)-1-(3-pyridyl)-1-butanone. Both compounds release formaldehyde *in vivo *after they have been modified by enzymes of the P450 family. While this shows the formation of formaldehyde-induced crosslinks *in vivo *for the first time, concentrations of free formaldehyde in the study were very much lower than those encountered in formaldehyde preservation of tissues, therefore apparently crosslinking some DNA bases while not preventing DNA extraction. The effective formaldehyde concentration during standard tissue fixation is considered to be much higher, and hence lead to much more extensive crosslinking.

The very nature of the crosslinks formed in the process of formaldehyde fixation seems to prevent the DNA from being extracted from the tissue. While we assume such crosslinks to be present, we have not yet been able to extract DNA with such modifications from the tissue and make it available for further analysis.

### III. Modeling DNA fragmentation

In order to better understand the observed fragmentation pattern of the moth DNA samples, we considered two main sources of fragmentation, simultaneous double-strand breaks and nicking, and applied simple mathematical models for the two processes. We make no assumptions as to the cause of nicks and strand breaks in this model, and do not account for changes in the rate of DNA degradation over the storage time.

These models were first motivated by our findings from the moth DNA samples. The change in average fragment size from 70 bp in seven-year-old samples to 45 bp in 40-year-old samples seemed surprisingly small, and the fragmentation in the youngest samples very high. We first used a simple approach to model fragmentation patterns resulting from double-strand breaks. Such a model can give some insight into the observed fragment sizes of the moth DNA, to be complemented by a model based on nicking, see below. We assumed a correlation for the size distribution of a DNA strand affected by a certain number of strand breaks per time as shown in equation 3:

$$l_{t}=\frac{l_{0}}{B\times t}$$

*l*_0_*: initial length of DNA*

B: number of strand breaks per day

t: time in days

*l*_*t*_*: average fragment size after time t*

**Figure 9:**
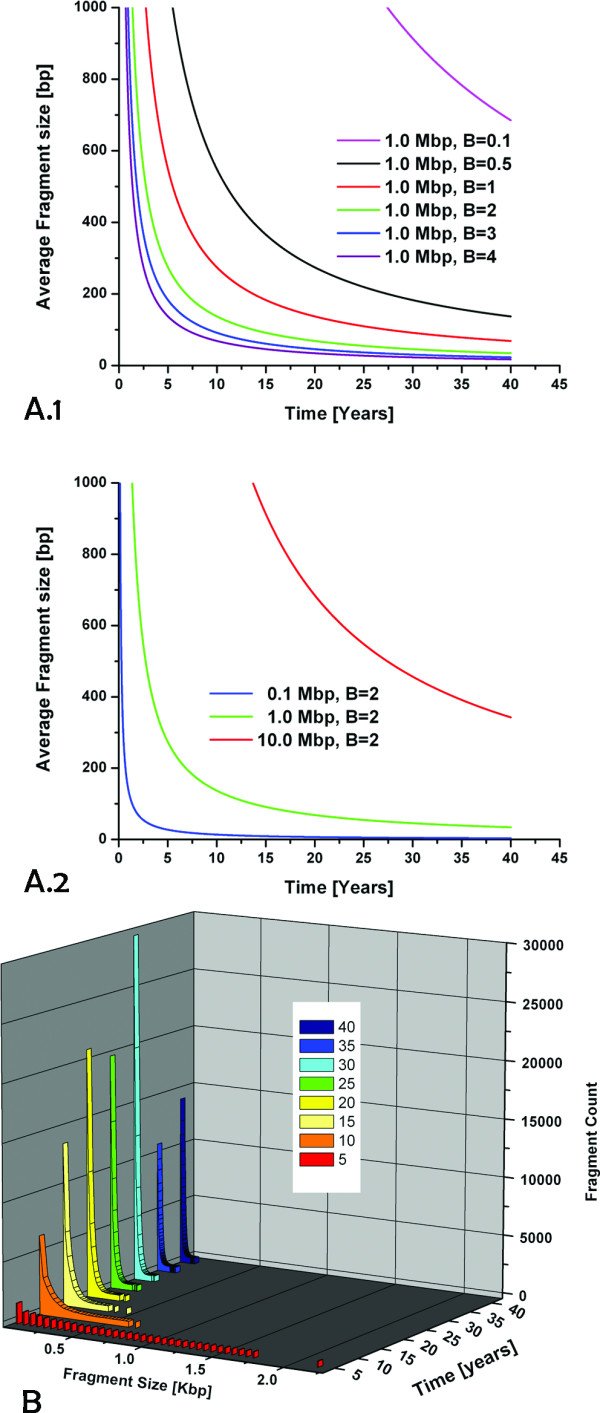
**Modeling of DNA fragmentation**. Two models of DNA fragmentation were analyzed. Model 1 is based on double-strand breaks. Panel A.1 shows the resulting average fragment sizes that occur over time for an initial fragment length of 1 Mbp, assuming different rates of strand breaks per day. Panel A.2 shows fragment sizes resulting from different initial lengths of DNA, for a rate of two strand breaks per day. Model 2 is based on nicking of single strands in double-stranded DNA. Panel B shows the distribution of fragments resulting from a starting size of 1 Mbp, at a rate of 18 nicks per day.

The resulting development of the average fragment size over a period of forty years is shown in Figure 9A. For a starting length of 1 Mbp, a fast drop in the first five years is followed by a much more gradual change. After reaching an average size of 200 bp after 7 years, it would take another 63 years for the average fragment size to go down to 20 bp, always assuming a constant rate of introduction of strand breaks. Accordingly, starting from 10 Mbp, it would take 700 years to get to 20 bp pieces. This model is an approximate reproduction of the observed change in fragment size as described above, in that we observe the same gradual change over an extended age range of samples, after an (assumed) initial fast drop.

Trying to fit this model to our data was not successful, primarily due to the lack of experimental data for very early stages of fragmentation. Also, double-strand breaks will not be the only source of DNA fragmentation, so that it may not be feasible to describe the experimental data based on this process alone.

Therefore, we modeled the accumulation of nicks, e.g., single-strand breaks, over time. Assuming that two nicks on opposing strands of DNA will lead to a double-strand break if they are separated by ten or fewer base pairs, we calculated the resulting distribution of fragment sizes and the change of this distribution over time. This can lead to a prediction of longer fragments of DNA in samples that are overall dominated by shorter fragments. This in turn may determine the feasibility of amplifications of larger sequence stretches from older samples.

The results are shown in Figure 9B. We assumed a rate of nicking of 18 nicks per day, based on previously reported estimates [37]. Starting from an initial size of 1 Mbp, the predicted fragment size drops rapidly and is below 2 Kbp after only five years even for the remaining larger fragments. After 15 years, most fragments are predicted to be smaller than 300 bp.

In combination, the two models of double-strand breaks and nicking highlight the scope and limitations associated with the amplification of DNA from stored and aged samples. The trends observed in the models correspond well with our findings from moth DNA samples. Fresh DNA samples pose practically no limit to the length of amplifiable DNA stretches, yet the amplifiable fragment length in preserved tissues declines very rapidly, and fragmentation appears to be the main reason for failure of amplification of longer sequences, more deleterious than the accumulation of specific base lesions.

Both models are based on the accumulation of a certain number of lesions after a given time and make no assumptions on the rate of introduction of such lesions. This rate may well be variable in real samples over time, depending on sample composition and storage/preservation conditions.

## Conclusion

We investigated the molecular properties of DNA samples extracted from museum specimens. DNA extracted from individual moth specimens stored between 5 and 40 years was subjected to fragment analysis and HPLC-MS analysis. We showed the degree and progress of fragmentation in these samples and corroborated the observed fragment sizes by two models of fragmentation. These findings may aid in the design of studies utilizing such samples, and help researchers to make educated guesses about the amplicon size that may reasonably be expected from a sample of a given age stored under comparable conditions. In particular, we found that a 500 bp amplicon can readily be obtained from samples up to ten years in age, whereas shorter sequences need to be targeted in older samples. With respect to using such samples to obtain DNA barcodes, a longer barcode sequence can only be concatenated from shorter subsequences of 100 bp or less.

Our investigation of DNA extraction and characterization of frog tissue preserved in ethanol or formaldehyde corroborates many findings reported previously by other researchers. Tissue lysis is the main obstacle in obtaining DNA from formaldehyde-exposed tissues. The DNA yield is low, but extractable DNA does not exhibit major base lesions, suggesting that crosslinked DNA was not extractable.

This study should guide future projects in a) the choice of preservation: Combined with a wealth of research in the past, our study reinforces that samples must not be exposed to formaldehyde if the contained DNA is to be utilized in any kind of downstream process; and b) the design of sequencing projects on stored insect and tissue samples, with regard to amplicon length and expected fragmentation.

## Abbreviations

LC-MS: Liquid-Chromatography Mass Spectrometry; HPLC: High Performance Liquid Chromatography; ESI: Electrospray Ionization; TOF: Time Of Flight; CE: Capillary Electrophoresis.

## Competing interests

The authors declare that they have no competing interests.

## Authors' contributions

JZ extracted DNA from frog specimens, ran PFGE and PCR experiments, analyzed the capillary electrophoresis data, performed the LC-MS analysis and drafted the manuscript. MH provided moth DNA samples. DB designed the sampling of frog tissues and collected frog specimens in the field. EC ran the capillary electrophoresis experiments. JP devised and implemented the program for fragmentation modeling. TE conceived of the study and participated in its ongoing design. All authors read, edited and approved the final manuscript.
